# Clustering evolving proteins into homologous families

**DOI:** 10.1186/1471-2105-14-120

**Published:** 2013-04-08

**Authors:** Cheong Xin Chan, Maisarah Mahbob, Mark A Ragan

**Affiliations:** 1 Institute for Molecular Bioscience, The University of Queensland, Brisbane, QLD, 4072, Australia; 2 Australian Research Council Centre of Excellence in Bioinformatics, Brisbane, QLD, 4072, Australia; 3 School of Chemistry and Molecular Biosciences, The University of Queensland, Brisbane, QLD, 4072, Australia

## Abstract

**Background:**

Clustering sequences into groups of putative homologs (families) is a critical first step in many areas of comparative biology and bioinformatics. The performance of clustering approaches in delineating biologically meaningful families depends strongly on characteristics of the data, including content bias and degree of divergence. New, highly scalable methods have recently been introduced to cluster the very large datasets being generated by next-generation sequencing technologies. However, there has been little systematic investigation of how characteristics of the data impact the performance of these approaches.

**Results:**

Using clusters from a manually curated dataset as reference, we examined the performance of a widely used graph-based Markov clustering algorithm (MCL) and a greedy heuristic approach (UCLUST) in delineating protein families coded by three sets of bacterial genomes of different G+C content. Both MCL and UCLUST generated clusters that are comparable to the reference sets at specific parameter settings, although UCLUST tends to under-cluster compositionally biased sequences (G+C content 33% and 66%). Using simulated data, we sought to assess the individual effects of sequence divergence, rate heterogeneity, and underlying G+C content. Performance decreased with increasing sequence divergence, decreasing among-site rate variation, and increasing G+C bias. Two MCL-based methods recovered the simulated families more accurately than did UCLUST. MCL using local alignment distances is more robust across the investigated range of sequence features than are greedy heuristics using distances based on global alignment.

**Conclusions:**

Our results demonstrate that sequence divergence, rate heterogeneity and content bias can individually and in combination affect the accuracy with which MCL and UCLUST can recover homologous protein families. For application to data that are more divergent, and exhibit higher among-site rate variation and/or content bias, MCL may often be the better choice, especially if computational resources are not limiting.

## Background

Homology is the basis of comparative biology [1], and recognising sets of homologous genes or proteins underlies much of modern bioscience including genome annotation, phylogenetic inference and studies of protein structure. Particularly in high-throughput applications, these molecules are usually arranged into putatively homologous sets based on sequence similarity. A greater degree of shared similarity (smaller distance) observed among a set of sequences relative to the others in the dataset indicates a likely homologous history.

In recent studies [2,3], clustering approaches have been loosely grouped into three classes based on their algorithmic design: hierarchical, greedy heuristic, and Bayesian. Hierarchical clustering approaches, e.g. ESPRIT-Tree [4], operate on pairwise distances (an estimate of pairwise relatedness) that are commonly generated by local alignment (e.g. using BLAST) and group sequences at a defined threshold of similarity. While computationally demanding of space and memory, greedy heuristic approaches such as CD-HIT [5] and UCLUST [6] are more scalable, in substantial part because they simultaneously compute pairwise similarity and group the sequences using a greedy global-alignment algorithm. CD-HIT has been adopted in major databases [7,8] to delineate protein families and reduce data redundancy, while UCLUST, which uses a fast global-alignment algorithm (UBLAST) that takes only best hits into consideration, can accurately delineate highly conserved protein sets [6]. In addition to these three categories, MCL [9], adopting probability and graph flow theory within a Markov matrix framework, has been widely adopted for delineating families in phylogenetic studies [10-14]. The performance of these approaches necessarily varies depending on features of the data, including content bias (i.e. G+C bias in genes, or the consequent bias in the proteins these genes encode), and the degree to which the sequences have diverged in the course of evolution. A recent benchmarking analysis [3] using both empirical and simulated data suggests that hierarchical approaches are more accurate in recovering sequence clusters than are greedy heuristics. Nevertheless the contribution of these features individually on the performance of clustering approaches remains unclear.

Here we assess the clustering performance of MCL in comparison to the fast, greedy heuristic clustering method, UCLUST. We approach this issue in two ways. First, we examine the performance of these two approaches in clustering empirical protein datasets from three sets of bacterial genomes at varied levels of G+C content: *Staphylococcus* at *∼* 33% G+C [15], *Mycobacterium* at *∼* 66% G+C ( http://www.tbdb.org/) and *Escherichia coli*/*Shigella* with *∼* 50% G+C [16]. Different clustering performance across these three datasets would therefore indicate, at least partially, the sensitivity of these approaches to G+C content of the genomes. Second, we generated sets of sequences by simulation on a tree, in order to assess individually the impact on clustering performance of sequence divergence, rate heterogeneity, and bias in G+C content.

## Results and discussion

We examined, respectively for MCL and UCLUST, the similarity between two sets of clusters (i.e. similarity of clustering assignments between two sets), as estimated by comparing all possible paired member assignments using the Adjusted Rand Index (*ARI*[17]), which ranges between −1 and 1. This measure, based on the agreement of cluster memberships between two sets of data, has been widely adopted to measure clustering accuracy, e.g. [18,19]. *ARI >* 0 indicates that the two sets share a number of identical clusters (*ARI* = 1 indicates identical clustering assignments between the two sets), *ARI* = 0 indicates the two sets do not agree exactly on any cluster memberships, and *ARI <* 0 indicates the deviation between the two sets is greater than expected by chance.

### Analysis of empirical data

Sets of protein sequences encoded by genomes of three bacterial genera were obtained from previously published work, or from public databases: 34066 proteins from 13 *Staphylococcus* genomes [15], 86393 proteins from 19 *Mycobacterium* genomes http://www.tbdb.org/ as of 5 December 2011, and 123136 proteins from 27 *Escherichia coli* and *Shigella* genomes [16]. As our reference cluster dataset we extracted all relevant clusters (i.e. clusters containing proteins from the genomes used in this study) from the manually curated protein cluster database FigFam [20], as available from the Pathosystems Resource Integration Center (PATRIC) website [21], http://www.patricbrc.org/. For MCL, we used both BLASTP and UBLAST to generate the distance matrices (see Methods for detail). The MCL results based on BLAST+ and UBLAST searches are designated BLAST+MCL and UBLAST+MCL respectively.

**Figure 1:**
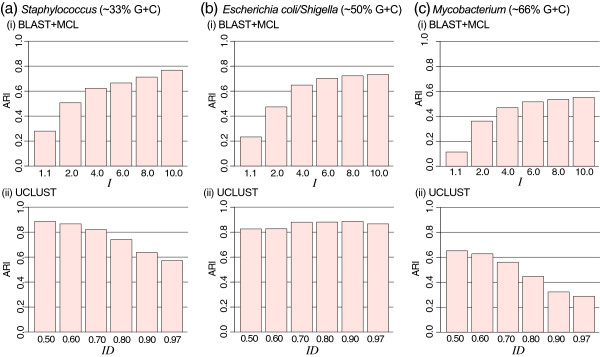
**Clustering accuracy of MCL and UCLUST on empirical data.** The *ARI* values observed in clustering of (**a**) *Staphylococcus* (low G+C *∼* 33%), (**b**) *Escherichia coli/Shigella* (moderate G+C *∼* 50%), and (**c**) *Mycobacterium* (high G+C *∼* 66%) proteins using (i) BLAST+MCL and (ii) UCLUST. See also Additional file 1: Figure S1.

Figure 1 shows the *ARI* for each comparison of all possible paired members between the generated and reference clusters, respectively for MCL (BLAST + MCL) and UCLUST clustering, across the relevant parameter settings, i.e. inflation parameter *I* for MCL and identity threshold *ID* for UCLUST. Results of UBLAST+MCL are not shown because they are highly similar to those of BLAST+MCL (see Additional file 1: Figure S1). In general, the clusters of *Escherichia coli*/*Shigella* and *Staphylococcus* generated by either BLAST + MCL or UCLUST are more similar to the reference (maximum *ARI* = 0.89 for UCLUST at *ID* 0.90 for *Escherichia coli*/*Shigella*), than are the clusters of *Mycobacterium* (maximum *ARI* = 0.65 for UCLUST at *ID* 0.50).

The number of clusters increases drastically with increasing *ID* in UCLUST (particularly of clusters of size *N <* 4), in comparison to MCL (both BLAST+ and UBLAST+MCL), in which the number of clusters remains similar across *I* (Additional file 1: Figure S1). When comparing cluster sizes, we focus on those of size *N ≥* 4 as these clusters are phylogenetically meaningful. For *Staphylococcus*, *Escherichia coli*/*Shigella* and *Mycobacterium*, the reference numbers of clusters (*N ≥* 4) are 2602, 5974 and 5429 respectively. The results closest to these, although not necessarily the most accurate (i.e. not the highest *ARI*), are those generated using BLAST+MCL with 2611 (*I* = 6.0, *ARI* = 0.67), 5302 (*I* = 10.0, *ARI* = 0.73), and 5433 (*I* = 8.0, *ARI* = 0.80), in comparison to 2663 (*ID* = 0.50, *ARI* = 0.88), 5797 (*ID* = 0.90, *ARI* = 0.89) and 5353 (*ID* = 0.80, *ARI* = 0.45) using UCLUST (Additional file 1: Figure S1).

We observed an increase in *ARI* as *I* increases in MCL across all data. A similar trend is observed in UCLUST for the *Escherichia coli*/*Shigella* data, in which *ARI* increases proportionately with *ID*, with the maximum achieved at *ID* 0.90. However, for *Mycobacterium* and *Staphylococcus* (data with G+C bias), a reverse trend is observed in UCLUST. In these instances, *ARI* decreases when higher *ID* threshold was applied. This trend can partly be explained by the lower within-cluster sequence similarity observed for the *Mycobacterium* and *Staphylococcus* dataset than for *Escherichia coli/Shigella* (Additional file 1: Figure S2). FigFam protein families of *Mycobacterium* (mean identity 51.3%, median 83.2%) and of *Staphylococcus* (mean identity 55.8%, median 77.5%) are more divergent (less similar) than those of *Escherichia coli/Shigella* (mean identity 63.9%, median 98.2%). Although such low within-cluster similarity (peaks in region of *<* 20% identity in Additional file 1: Figure S2) could be explained by single-member clusters (within-cluster identity 0%), such divergence could partly be explained by compositional biases in these genomes. Therefore a lower *ID* threshold in UCLUST appears to perform better in these cases. We expect clustering performance to increase as annotation of these proteins improves as more genome data become available.

These results, if general, indicate that the greedy heuristics approach tends to under-cluster compositionally biased sequences. This is not due to the UBLAST algorithm implemented in UCLUST, as MCL generates about the same numbers of clusters when we take bit scores from UBLAST (compare BLAST+MCL versus UBLAST+MCL). In addition to G+C bias, other evolutionary parameters e.g. among-site variation of substitution rates and/or convergence could also have contributed to our result. Highly similar sequences play to the strength of global alignment (as implemented in UCLUST) more than to local alignment (BLAST), as shown by the high *ARI* values in Figure 1. Our MCL clustering results shown in Figure 1 are based on the BLAST *e*-value cut-off at 10^*−*3^. As we applied more-stringent thresholds at 10^*−*10^ and 10^*−*25^, we observe higher clustering accuracy in MCL (Additional file 1: Figure S3). For instance, in the case of *Staphylococcus*, MCL clustering at *I* = 10.0 yielded *ARI* 0.88 (the highest *ARI* for UCLUST is 0.89 at *ID* = 0.50) based on BLAST *e ≤* 10^*−*25^, in comparison to 0.79 and 0.77 (*I* = 10.0) at *e ≤* 10^*−*10^ and 10^*−*3^, respectively. Our results support the notion that selecting an appropriate threshold of similarity measures is key in optimising clustering performance [22]. Because with empirical data it is difficult to isolate or distinguish individual causative factors, we next simulated the evolution of families of homologous proteins under controlled settings of branch length (sequence divergence), rate heterogeneity and compositional bias.

### Analysis of simulated data

**Figure 2:**
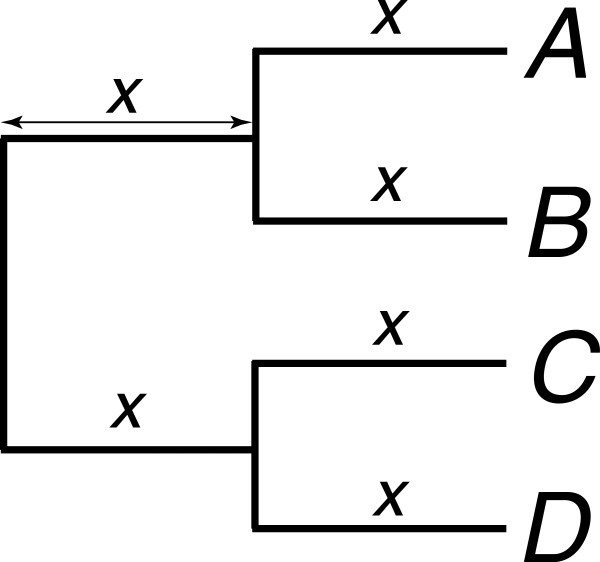
**Tree topology used in the simulation of sequence data.** The topology depicting the evolutionary relationship among sequences ***A***, ***B***, ***C*** and ***D*** in the simulated protein family; *x* represents the length of each internal branch (unit: number of substitutions per site).

Using simulated data of protein sequences, we assessed the clustering performance based on three individual evolutionary aspects: (a) sequence divergence, (b) among-site rate heterogeneity, and (c) G+C bias. All simulated datasets were generated using evolver as implemented in PAML 4.5 [23]. For each designated parameter setting, we generated 2500 protein families each of size *N* = 4 (sequences *A*-*D*) and of length 800 amino acids, by simulation on an unrooted symmetrical tree (Figure 2), on which all internal branches (*x*) are of the same length.

The *ARI* described above measures clustering accuracy in these methods, but does not provide any insights into cluster numbers and sizes, which could be useful in understanding the exact nature of mis-clustering. As we know the exact cluster size and numbers in our simulated data, here we adopted an additional, independent measure for performance accuracy based on cluster sizes. Given *N*_*C*_ = mean *N* among the resulting clusters, and *N*_*R*_ = mean *N* observed in the reference set, we denote *δ* as a measure of fold difference of the average cluster size against the reference: *δ* = *N*_*C*_/*N*_*R*_. Here we follow Clark et al. [24] in defining the instances of over-clustering and under-clustering. Over-clustering is observed when *δ >* 1 (i.e., the average cluster size is larger than in the reference), and under-clustering when *δ <* 1 (i.e., the average cluster size is smaller than in the reference). We apply *δ* to all clusters, i.e. *δ* is simply the overall average cluster size divided by the known size *N* = 4. If our clustering methods perform perfectly, we expect to recover 2500 protein sets each of size *N* = 4 (*δ* = 1 and *ARI* = 1). We might also anticipate that as sequences grow more divergent, patchy or biased, clustering methods may become less efficient in grouping them correctly within families, and/or in distinguishing these families from each other. The results we present here are averages across five replicates. Given that the minimum cluster size is 1, the minimum *δ* across these instances is 1/4 = 0.25. For all the results described below, all instances where *δ* = 1 yielded sets that are identical to the reference (*ARI* = 1).

#### Sequence divergence

To assess the effect of sequence divergence, we simulated the sequence data on an unrooted tree (Figure 2), progressively set the internal branch length *x* = 0.10, 0.25, 0.50, 0.75 and 1.00 substitutions per site, and used a discrete approximation of the gamma distribution (shape parameter *α* = 1.0, 8 categories).

**Figure 3:**
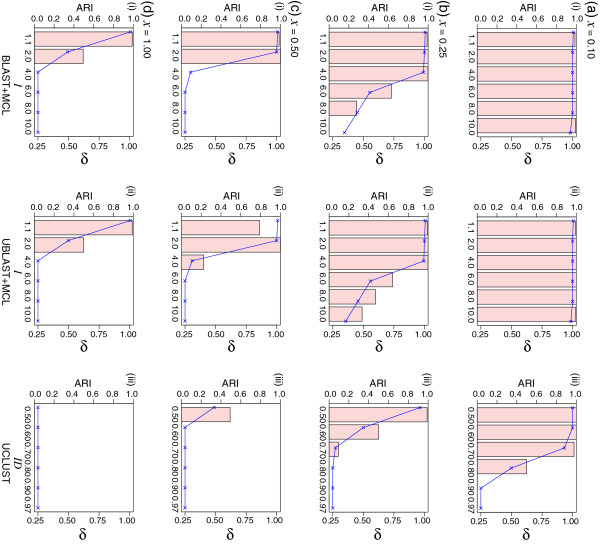
**The effect of sequence divergence on clustering accuracy.** In each panel, the bar chart shows the *ARI* values (Y-axis on the left) observed from clustering of the data simulated at various branch length (*x*) of tree topology shown in Figure 2, with *x* set at (**a**) 0.10, (**b**) 0.25, (**c**) 0.50, and (**d**) 1.00 substitutions per site, using (i) BLAST+MCL, (ii) UBLAST+MCL, and (iii) UCLUST, across the specific parameter settings (X-axis on each panel: *I* for MCL, *ID* for UCLUST). All numbers shown are averaged across five replicates in each instance. Standard deviation from the mean in each case (not shown) is *<* 0.02. The *δ* values are plotted within the same panel (Y-axis on the right). See also Additional file 1: Figure S4.

As divergence increases from 0.1 to 1.0 mean substitutions per site (branch length *x* in Figure 2), we observe a decrease in the clustering performance of all three methods (Figure 3). The number of generated clusters is shown in Additional file 1: Figure S4. At *x* = 0.1 substitutions per site (Figure 3a), the 2500 homologous sets were perfectly recovered by UCLUST at *ID ≤* 0.60 (*ARI* = 1.00, *δ* = 1.00), and almost perfectly at *ID* = 0.70 (2499 clusters, *ARI* = 0.98, *δ* = 0.93). UCLUST, however, failed to recover any clusters at *ID ≥* 0.90 (10000 clusters, *ARI* = 0.00, *δ* = 0.25). In contrast, both MCL-based methods recovered all 2500 protein sets (*ARI*, *δ* = 1.00) at settings of *I* between 2.0 and 6.0. BLAST+MCL and UBLAST+MCL tend to over-cluster slightly at *I* = 1.1, yielding 2477 (*ARI* = 0.99, *δ* = 1.01) and 2484 clusters (*ARI* = 0.99, *δ* = 1.01) respectively, and to under-cluster slightly at *I* = 10.0, generating 2536 (*ARI* = 0.99, *δ* = 0.98) and 2535 (*ARI* = 0.99, *δ* = 0.99) clusters respectively. As *x* increases from 0.25 to 1.0 (Figures 3b to 3d) the 2500 protein sets were still recovered using either of the two MCL approaches at low *I* settings (particularly at *I* = 1.1), whereas UCLUST usually failed to recover any families of *N ≥* 4 whatsoever when *x ≥* 0.25. As shown in Additional file 1: Figure S5a, incrementing *x* from 0.10 to 1.0 resulted in the gradual decrease of average within-cluster sequence similarity from 76.6% to 31.0%.

These results suggest that local alignment-based approaches using MCL are more effective in recovering homologous protein families than the global alignment-based method, when sequences are (or appear to be) more evolutionarily distant from each other. Some proteins, although sharing little sequence similarity, are known to be structural homologs, i.e. sharing high similarity in folding structure due to a common ancestral origin [25,26]. In these instances, clustering of these highly divergent sequences (but with conserved structural features) into a single homologous family would be desirable. When the level of sequence divergence is the only determining factor for the protein clusters, MCL with *I ≤* 2 appears to be a better option than UCLUST.

#### Among-site rate heterogeneity

We modelled rate heterogeneity (distribution of among-site rate variation) across-sequence under the discrete approximation of the (continuous) gamma distribution, with the shape of the distribution determined by the *α* parameter. A small value (*α ≤* 1) implies that substitution differs greatly across sites, e.g. a few sites have diversified quickly while the others little or not at all. This condition is analogous to having multiple conserved domains within a protein sequence. A large value (*α >* 1), on the other hand, indicates that most sites have diversified at about the same rate. To assess the effect of rate heterogeneity we fixed *x* = 0.10 and progressively set *α* = 0.5, 1.0 and 2.0 under the same 8-category discrete gamma distribution.

**Figure 4:**
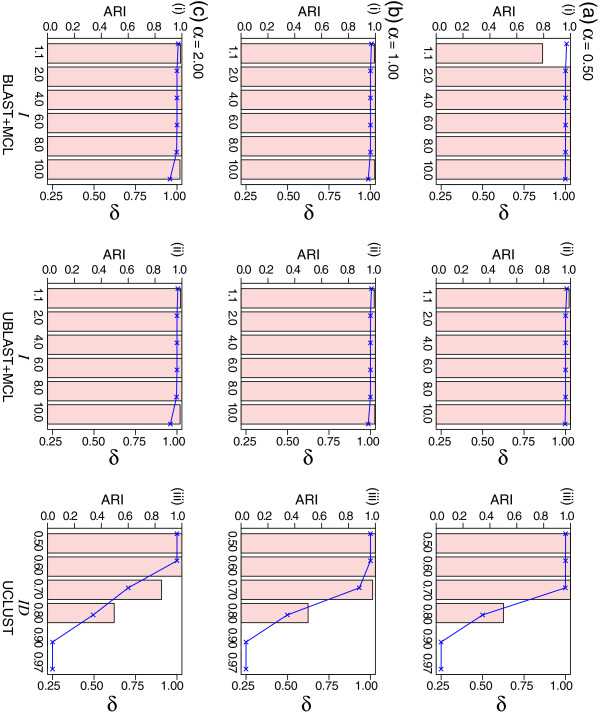
**The effect of rate heterogeneity on clustering accuracy.** In each panel, the bar chart shows the *ARI* values (Y-axis on the left) observed from clustering of the data simulated using *α* values (the shape parameter) of gamma distribution set at (**a**) 0.5, (**b**) 1.0, and (**c**) 2.0, using (i) BLAST+MCL, (ii) UBLAST+MCL, and (iii) UCLUST, across the specific parameter settings (X-axis on each panel: *I* for MCL, *ID* for UCLUST). All numbers shown are averaged across five replicates in each instance. Standard deviation from the mean in each cases (not shown) is *<* 0.02. The *δ* values are plotted within the same panel (Y-axis on the right). See also Additional file 1: Figure S6.

Figure 4 shows how the three clustering methods perform when rates are heterogeneous within a low, uniform sequence divergence (*x* = 0.10 in Figure 2). The number of generated clusters is shown in Additional file 1: Figure S6. Interestingly for the two MCL approaches, as *α* increases from 0.5 to 2, the 2500 protein sets (*ARI* = 1.00, *δ* = 1.00) were recovered in almost all cases across all inflation parameter values, except at *I ≥* 8.0. Even at *I* = 10.0 and *α* = 2, MCL recovered 2612 clusters (*ARI* = 0.99, *δ* = 0.96). Interestingly, for BLAST+MCL (*α* = 0.5) at *I* = 1.1, although *δ* = 1.01, the observed *ARI* is 0.79, suggesting clustering errors. In comparison, UCLUST became much less efficient in recovering the correct protein sets as *α* was increased. For instance, at *ID* = 0.70, the number of protein clusters increases, and performance accuracy decreases, in proportion to *α*, from 2501 (*ARI* = 1.00, *δ* = 1.00) to 2672 (*ARI* = 0.98, *δ* = 0.94) to 3554 (*ARI* = 0.85, *δ* = 0.70) respectively at *α* = 0.5, *α* = 1.0 and *α* = 2.0. Families of *N* = 4 could be recognised at lenient *ID* thresholds (0.50 and 0.60) but thresholds of 0.80, 0.90 and 0.97 (the default in UCLUST) were too stringent. As shown in Additional file 1: Figure S5b), changes in *α* do not result in significant changes in within-cluster sequence similarity (mean ranging between 75.2 and 78.9% in the three cases).

By itself, increased across-sites rate heterogeneity does not affect clustering accuracy as much as does greater sequence divergence. All three clustering approaches recovered the correct number of families more efficiently when rate heterogeneity was high (*α ≤* 1) than when it was low (*α >* 1). The local alignment-based approach using MCL is more robust than the greedy heuristic of UCLUST to heterogeneity of rate across sites. We examined the data across other *α* values and found negligible difference, i.e. cases of *α* = 5 and 10 are similar to the case of *α* = 2, while the case of *α* = 0.1 is similar to the case of *α* = 0.5.

#### Compositional bias of coding sequences

**Figure 5:**
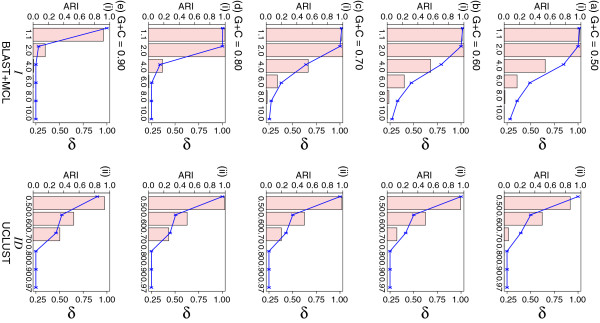
**The effect of G+C content of the coding DNA sequences on clustering accuracy.** In each panel, the bar chart shows the *ARI* values (Y-axis on the left) observed from clustering of the data simulated using G+C proportion at (**a**) 0.5, (**b**) 0.6, (**c**) 0.7, (**d**) 0.8, and (**e**) 0.9, using (i) BLAST+MCL and (ii) UCLUST, across the specific parameter settings (X-axis on each panel: *I* for MCL, *ID* for UCLUST). All numbers shown are averaged across five replicates in each instance. Standard deviation from the mean in each case (not shown) is *<* 0.02. The *δ* values are plotted within the same panel (Y-axis on the right). See also Additional file 1: Figure S7.

For this part of analysis, we generated (i.e. translated) protein sequences from simulated DNA sequences at varying levels of G+C composition (see Methods for details). With both MCL and UCLUST, clustering accuracy of the protein sequences falls off slightly as the underlying simulated DNA sequences become more compositionally biased (Figure 5). The number of generated clusters is shown in Additional file 1: Figure S7. In this analysis, the lack of filtering for low-complexity sequences in UBLAST resulted in a huge number of sequence hits among the high G+C data (thus little variation in the resulting protein sequences), and therefore a pairwise matrix that is too large for practical clustering using MCL. As such, only BLAST+MCL and UCLUST results are shown. From 50-80% G+C almost all families are recovered by MCL at *I* = 1.1 and 2.0 (Figure 5, panels of column i); for example, at *I* = 2.0 BLAST+MCL recovers 2500 families (*ARI* = 1.00, *δ* = 1.00) at 70% G+C and 2501 (*ARI* = 0.99, *δ* = 1.00) at 80%, but 8972 (*ARI* = 0.16, *δ* = 0.28) at 90% G+C. At *I >* 2.0, however, BLAST+MCL over-clusters; e.g. at *I* = 4.0, BLAST+MCL recovers 1838, 1101 and 50 sets of *N* = 4 at 60%, 70% and 80% G+C (*ARI* = 0.57, 0.55 and 0.18 respectively).

For UCLUST, sequence identity settings *ID >* 0.50 appear to be too stringent, as few sets of *N* = 4 were recovered (e.g. at *ID* = 0.60, where both *ARI* and *δ* approximate 0.50 at 80% and 90% G+C). Interestingly, at the most-extreme G+C content considered here (90%), BLAST+MCL at *I* = 1.1 recognised 2503 clusters (*ARI* = 0.92, *δ* = 0.99), of which 2498 have *N* = 4; in comparison, UCLUST at *ID* = 0.50 found 2784 clusters (*ARI* = 0.93, *δ* = 0.90), of which 2216 are of *N* = 4. The variation of G+C proportion across these simulated DNA sequences does not cause significant changes in pairwise sequence similarity within protein clusters (ranging between 58.6 and 64.6% with broader distribution at 90% G+C; see Additional file 1: Figure S5c), explaining why our results did not drastically change, particularly in UCLUST (Figure 5 and Additional file 1: Figure S7). In order to assess whether our simulated conditions in this experiment are intrinsically unfavourable to UCLUST in general (where no invariant sites were simulated), we examined the performance of UCLUST across instances of 50% G+C with an increasing proportion of invariant sites (*inv*) in the sequences, from 0.1 to 0.9, as shown in panels (a) to (i) in Additional file 1: Figure S8. Panel (j) in the same figure shows the average within-cluster pairwise sequence similarity of each of these cases. Evidently, as the proportion of invariant sites across the sequences increases, the efficiency of UCLUST in correctly identifying the four-member clusters increases. For instance, at *ID* = 0.7, UCLUST recovered almost 2500 clusters of *N* = 4 when *inv >* 0.7 (average within-cluster sequence similarity *>* 70% in Additional file 1: Figure S8j. Therefore, proportion of invariant sites (and thus sequence similarity) remains a key factor influencing the performance of UCLUST. Such conserved regions and invariant sites across sequences are expected in empirical data.

G+C bias inevitably influences codon usage in protein translation [27,28]. Given that changes at the third codon position (compared to those at first or second position) are more likely to be cryptic, the usage of codons ending in G or C is expected to increase with G+C bias. This was indeed observed in a recent study of various prokaryote and eukaryote genomes [29], except for two codons. The same study demonstrates that usage of some codons is non-linear to G+C bias. Whereas the different levels of G+C content causes little difference in the accuracies of the two approaches in clustering the resulting protein sequences, MCL is more robust to such fluctuation than UCLUST, as observed by number of protein sets of *N <* 4 (gray bars across the panels in column ii; Additional file 1: Figure S7). To analyse the effect of G+C bias, we allowed our simulated DNAs only a low degree of divergence (*x* = 0.1 in Figure 2). At the most-extreme G+C content we examined (90%) there is much-reduced scope for sequences (both DNA and the encoding proteins) to be recognisably different from each other, whether viewed locally or globally. As such, our observation that both MCL and UCLUST failed to recover the known number of families across most parameter-value settings is not surprising. Fortunately, few empirical data are biased to this degree.

The MCL-based methods are computationally more expensive than UCLUST. The most time-consuming step is the generation of the matrix of sequence relatedness using all-versus-all BLAST. For this study, all computation was done using a 640-node Linux high-performance computer cluster (each node consisting of 8 cores with 8GB memory, i.e. 2 × Quad-core AMD Opteron 2356 2.3 GHz). For a set of 10000 protein sequences, the BLAST analysis on a single CPU required 250–300 MB of memory and took ca 1 to 12 hours depending on sequence variation within the set, with analysis of protein sequences generated at 90% G+C bias requiring the most time and memory.

## Conclusions

We assessed the performance accuracy of two clustering approaches designed for different purposes. UCLUST was designed for *de novo* clustering of large datasets (e.g. sequence reads) to reduce data redundancy and size, while MCL is commonly used to delineate homologous sequence sets. The former is usually done at higher level of sequence similarities than the latter. Our study demonstrates that evolutionary aspects other than sequence divergence, e.g. among-site rate heterogeneity and G+C content bias, affect the clustering performance of these two approaches. We have demonstrated that sequence divergence, rate heterogeneity and content bias can individually and in combination affect the accuracy with which MCL and greedy heuristic algorithms can recover homologous protein families. We found the impact to be broader and more severe on the heuristics-based UCLUST than on two variants of MCL implementations. The simple global percent identity among sequences adopted in the former may be advantageous when sets of proteins or ribosomal RNAs [4] are highly similar or contain overlapping or redundant regions (e.g. sequencing reads), or when extreme scalability is required, as the clustering of these sequences is computationally more tractable than MCL. For application to data that are more divergent, and exhibit higher among-site rate variation and/or content bias, MCL may often be the better choice, especially if computational resources are not limiting.

## Methods

### Simulation of protein families

All simulated datasets were generated using evolver as implemented in PAML 4.5 [23]. For each designated parameter setting, we generated 2500 protein families each of size *N* = 4 (sequences *A*-*D*) and of length 800 amino acids, by simulation on an unrooted symmetrical tree (Figure 2) on which all internal branches (*x*) are of the same length. To assess the effect of sequence divergence, we progressively set *x* = 0.10, 0.25, 0.50, 0.75 and 1.00 substitutions per site, and used a discrete approximation of the gamma distribution (shape parameter *α* = 1.0, 8 categories). To assess the effect of rate heterogeneity we fixed *x* = 0.10 and progressively set *α* = 0.5, 1.0 and 2.0 under the same 8-category discrete gamma distribution. For protein-sequence simulations we used the WAG substitution model [30].

To assess the effect of G+C content on clustering performance, simulation was similarly carried out at the nucleotide level for 2500 families, each of *N* = 4 and length = 2400 bases, with G+C proportion progressively at 0.5, 0.6, 0.7, 0.8 and 0.9, under the REV substitution model and a discrete gamma distribution (*α* = 1.0, 8 categories) but with *x* fixed at 0.1. We consider only cases of G+C proportion *>* 0.5 because sequences with G+C proportion *>* 0.5 are equivalent to sequences with A+T proportion *<* 0.5; these two instances (e.g. G+C 0.8 versus A+T 0.2, and the reverse situation of G+C 0.2) would have similar, if not identical, effects on our analysis. The nucleotide sequences were translated into protein sequences in frame +1. For stop codons (TAG, TAA and TGA), if present in a protein-coding region, the thymine residue was arbitrarily replaced with adenine to avoid interruption in protein translation (each protein consists of 800 amino acid residues). These stop codons were randomly distributed across all sequences and columns, therefore do not contribute to any clustering biases. To assess the impact of invariant site proportions on the performance of UCLUST, datasets with each specific proportion of invariant sites were simulated using INDELible [31], a more-flexible successor to PAML’s evolver. Across all simulated data, five replicates were generated for each parameter setting.

### Clustering using MCL

For each dataset, a distance matrix was first generated using an all-versus-all BLAST approach as described by Harlow et al. [10]. For this purpose we used BLASTP as implemented in NCBI BLAST+ version 2.2.25, and kept all matches with *e ≤*10^*−*3^. In parallel we also used UBLAST as implemented in USEARCH version 5.1.221 [6] as an alternative local alignment method to generate the distance matrix, using the option *−−*nousort (all hits, not only the top hit, were kept). Relatedness between sequences *a* and *b* (*R*_*ab*_) is based on their shared similarity, as observed in alignment bit scores: a normalised bit score (*B*) was first determined for each of *a* and *b*, in which *B*_*a*_ = score of *a* hitting *b* over the score of *a* hitting itself (*S*_*ab*_/*S*_*aa*_), and *B*_*b*_ = score of *b* hitting *a*, divided by the score of *b* hitting itself (*S*_*ba*_/*S*_*bb*_). *R*_*ab*_ is then defined as max(*B*_*a*_*, B*_*b*_). This definition of sequence relatedness has been described in a number of empirical studies [11,32-34], in which greater *R*_*ab*_ represents greater shared similarity (i.e. shorter distance) between the sequences. This matrix was used as input to MCL, with the inflation parameter *I* set at 1.1, 2.0, 4.0, 6.0, 8.0 or 10.0. The MCL results based on BLAST+ and UBLAST searches are designated BLAST+MCL and UBLAST+MCL respectively.

### Greedy heuristic clustering using UCLUST

We carried out UCLUST [6] using USEARCH version 5.1.221 (http://www.drive5.com/usearch/) with minimum proportion of identity of matches (*−*id) set at 0.5, 0.6, 0.7, 0.8, 0.9 or 0.97 (default). For datasets that consist of sequences of varied lengths, sequences were sorted by length in descending order (*−*sort option in USEARCH) prior to clustering, as required in the implementation of the program.

### Assessment of clustering performance

The *ARI* values [17] are calculated using the pdfCluster package [35] in R. For the analysis of simulated data, the *δ* values are derived from mean *N* among the resulting clusters (*N*_*C*_) divided by mean *N* observed in the reference set (*N*_*R*_) in each comparison. Within-cluster similarity is determined based on average pairwise similarity between each sequence in a cluster to the centroid sequence (i.e. the most-representative sequence) of the same cluster; pairwise identity is derived from BLAST (*e ≤* 10^*−*3^). The centroid sequence of a cluster is identified based on the sequence that yielded the single highest bit score across all pairwise comparisons within the cluster. Between-cluster similarity is calculated based on percent identity observed for all possible pairwise comparisons of these centroid sequences.

## Competing interests

The authors declared that they have no competing interests.

## Authors’ contributions

CXC and MAR conceived the study and designed the experiments. CXC and MM conducted the experiments. CXC, MM and MAR analysed and interpreted the results. CXC prepared the manuscript. All authors read and approved the final manuscript.

## Supplementary Material

Additional file 1: Figure S1 The number of clusters generated from proteins of (a) *Staphylococcus*, (b) *Escherichia coli/Shigella* and (c) *Mycobacterium* using (i) BLAST+MCL, (ii) UBLAST+MCL, and (iii) UCLUST. The number of clusters observed in the reference set (R) is shown at far left at each panel for comparison. The proportion of clusters with size *N* ≥ 4 is shown in red in each bar. **Figure S2.** Density histograms of within-cluster sequence similarities across the three bacteral protein datasets of *Escherichia coli/Shigella*, *Mycobacterium* and *Staphylococcus.* Histogram for between-cluster sequence similarities is not shown because almost all (> 99.89%) of between-cluster comparisons yielded no significant similarity. **Figure S3.** Clustering accuracy of BLAST+MCL across different *e*-value thresholds in BLAST and inflation parameter *I* in MCL for the proteins of (a) *Staphylococcus*, (b) *Escherichia coli/Shigella* and (c) *Mycobacterium*. **Figure S4.** Number of clusters generated across simulated dataset of various divergence levels. Data are shown for different branch lengths on a tree (*x* in Figure 2) at (a) 0.10, (b) 0.25, (c) 0.50 and (d) 1.00, for (i) BLAST+MCL (ii) UBLAST+MCL and (iii) UCLUST, across the specific parameter settings (*I* for MCL; *ID* for UCLUST). The proportion of clusters with size *N* ≥ 4 is shown in red bars. **Figure S5.** Density histograms of within-cluster sequence similarities across all simulated dataset at various levels of (a) sequence divergence (branch length *x* in Figure 2), (b) among-site rate heterogeneity (*α* in gamma distribution) and (c) compositional biases (G+C proportion). Histogram for between-cluster sequence similarities is not shown because almost all (> 99.99%) of between-cluster comparisons yielded no significant similarity. **Figure S6.** Number of clusters generated across simulated dataset of various rates of heterogeneity. Shown for alpha (*α*) value in gamma distribution at (a) 0.50, (b) 1.00 and (c) 1.00, for (i) BLAST+MCL (ii) UBLAST+MCL and (iii) UCLUST, across the specific parameter settings (*I* for MCL; *ID* for UCLUST). The proportion of clusters with size *N* ≥ 4 is shown in red bars. **Figure S7.** Number of clusters generated across simulated dataset of various G+C portions. Shown for G+C portion at (a) 0.50, (b) 0.60, (c) 0.70, (d) 0.80 and (e) 0.90, for (i) BLAST+MCL and (ii) UCLUST across the specific parameter settings (*I* for MCL; *ID* for UCLUST). The proportion of clusters with size *N* ≥ 4 is shown in red bars. **Figure S8.** Clustering accuracy of UCLUST across proportion of invariant sites in simulated dataset at 50% G+C. Results are shown for proportion of invariant sites from 0.1 through 0.9 in panels (a) through (i). In each of these panels, the bar chart shows the number of clusters (Y-axis on the left) across different *ID* parameters. All numbers shown are averaged across five replicates in each instance, and the error bars indicate standard deviation from the mean. The proportion of clusters with *N ≥ 4* is shown in red in each bar, and the δ values are plotted within the same panel (Y-axis on the right). Panel (j) shows average within-cluster pairwise sequence similarity of each of these cases. (PDF 2448 kb) Click here for file
