# Supplementary material for: Clustering evolving proteins into homologous families
Source: BMC Bioinformatics. 2013 Apr 8;14:120. doi: 10.1186/1471-2105-14-120 (PMC3637521; doi:10.1186/1471-2105-14-120)
Supplement: Additional file 1: Figure S1 — The number of clusters generated from proteins of (a) Staphylococcus, (b) Escherichia coli/Shigella and (c) Mycobacterium using (i) BLAST+MCL, (ii) UBLAST+MCL, and (iii) UCLUST. The number of clusters observed in the reference set (R) is shown at far left at each panel for comparison. The proportion of clusters with size N ≥ 4 is shown in red in each bar. Figure S2. Density histograms of within-cluster sequence similarities across the three bacteral protein datasets of Escherichia coli/Shigella, Mycobacterium and Staphylococcus. Histogram for between-cluster sequence similarities is not shown because almost all (> 99.89%) of between-cluster comparisons yielded no significant similarity. Figure S3. Clustering accuracy of BLAST+MCL across different e-value thresholds in BLAST and inflation parameter I in MCL for the proteins of (a) Staphylococcus, (b) Escherichia coli/Shigella and (c) Mycobacterium. Figure S4. Number of clusters generated across simulated dataset of various divergence levels. Data are shown for different branch lengths on a tree (x in Figure 2) at (a) 0.10, (b) 0.25, (c) 0.50 and (d) 1.00, for (i) BLAST+MCL (ii) UBLAST+MCL and (iii) UCLUST, across the specific parameter settings (I for MCL; ID for UCLUST). The proportion of clusters with size N ≥ 4 is shown in red bars. Figure S5. Density histograms of within-cluster sequence similarities across all simulated dataset at various levels of (a) sequence divergence (branch length x in Figure 2), (b) among-site rate heterogeneity (α in gamma distribution) and (c) compositional biases (G+C proportion). Histogram for between-cluster sequence similarities is not shown because almost all (> 99.99%) of between-cluster comparisons yielded no significant similarity. Figure S6. Number of clusters generated across simulated dataset of various rates of heterogeneity. Shown for alpha (α) value in gamma distribution at (a) 0.50, (b) 1.00 and (c) 1.00, for (i) BLAST+MCL (ii) UBLAST+MCL and (iii) UCLUST, across the spec [file 1471-2105-14-120-S1.pdf]

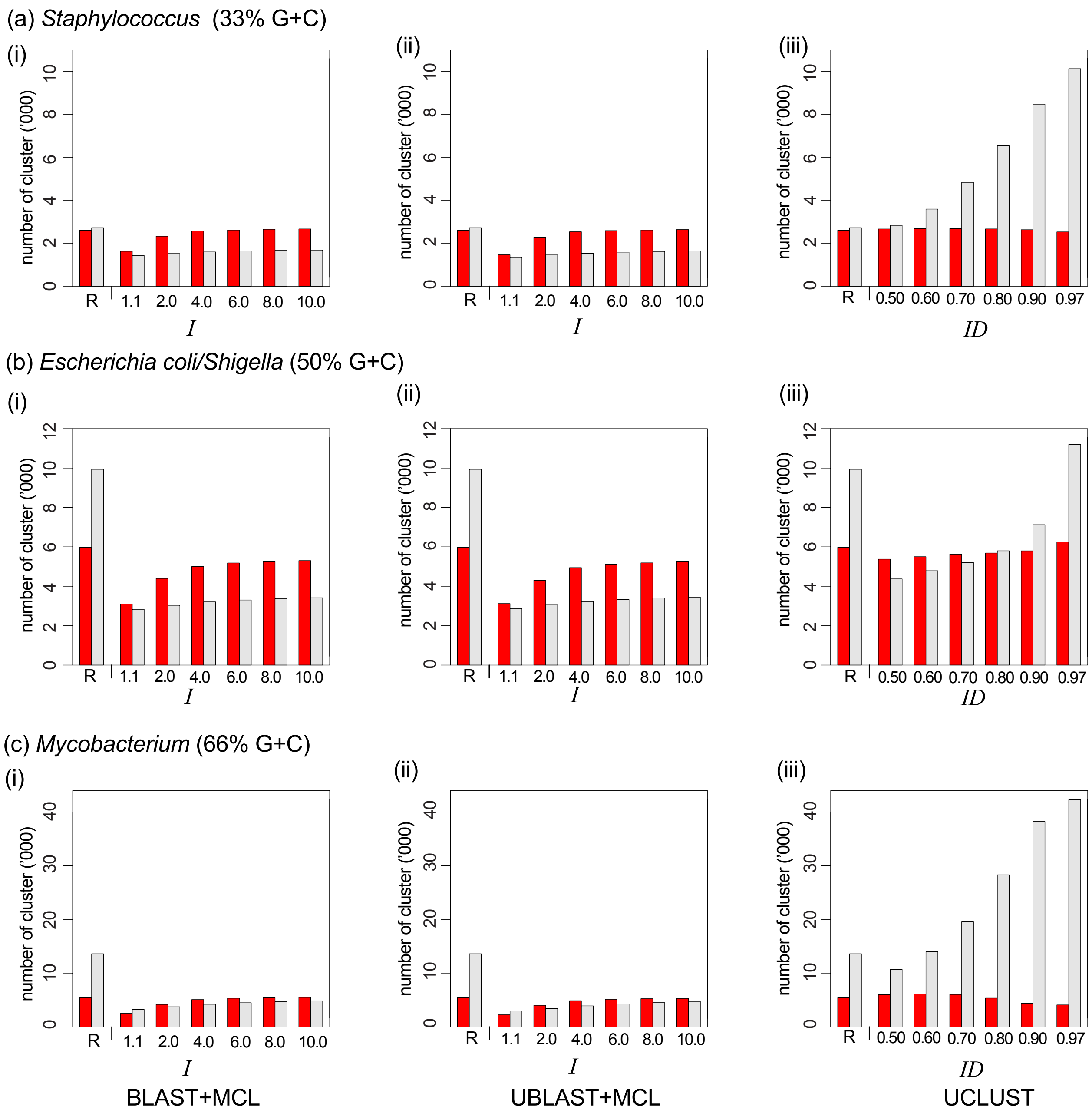

Figure S1. The number of clusters generated from proteins of (a) *Staphylococcus*, (b) *Escherichia coli/Shigella* and (c) *Mycobacterium* using (i) BLAST+MCL, (ii) UBLAST+MCL, and (iii) UCLUST. The number of clusters observed in the reference set (R) is shown at far left at each panel for comparison. The proportion of clusters with size $N \geq 4$ is shown in red in each bar.

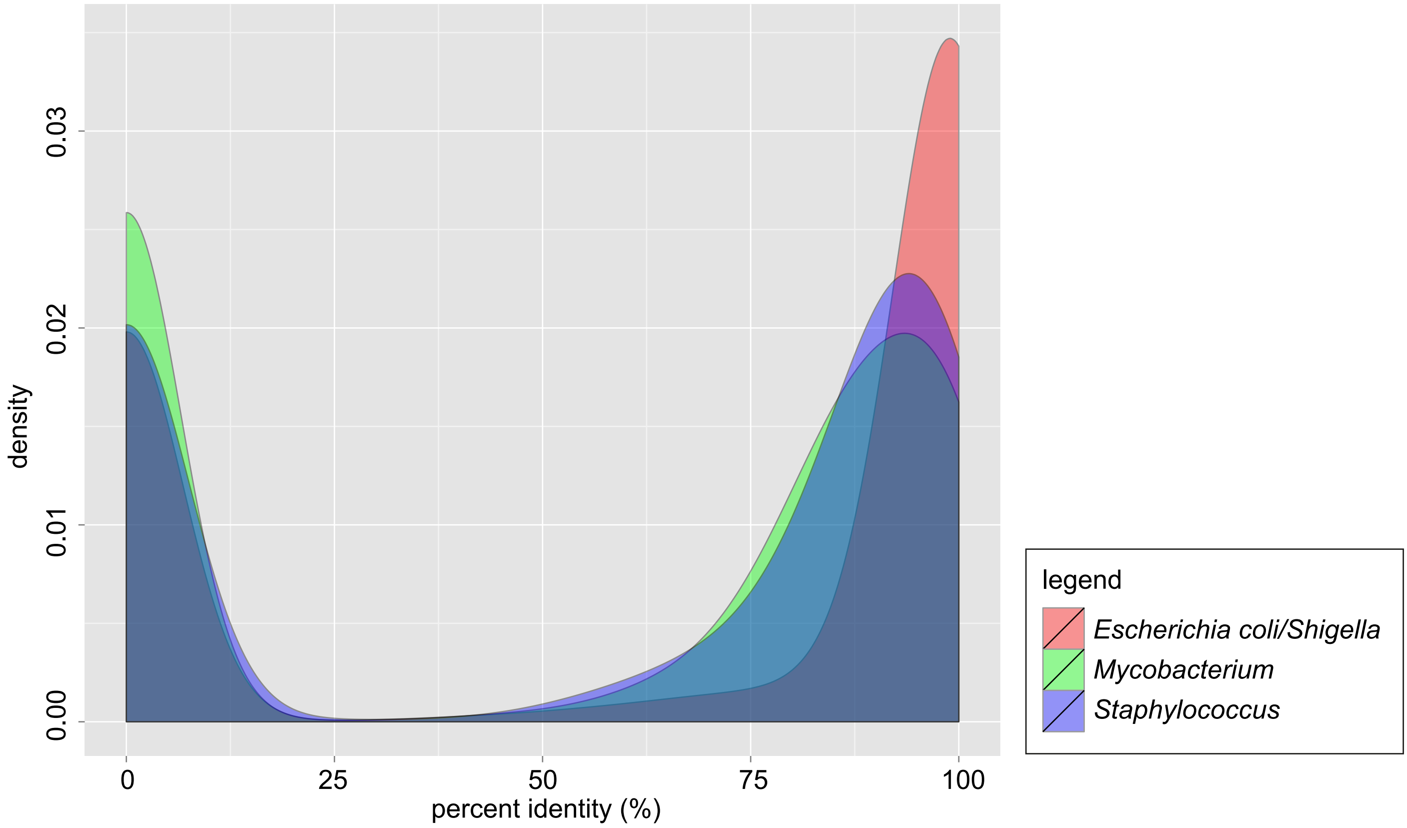

Figure S2. Density histograms of within-cluster sequence similarities across the three bacteral protein datasets of *Escherichia coli/Shigella*, *Mycobacterium* and *Staphylococcus.* Histogram for between-cluster sequence similarities is not shown because almost all (>99.89%) of between-cluster comparisons yielded no significant similarity.

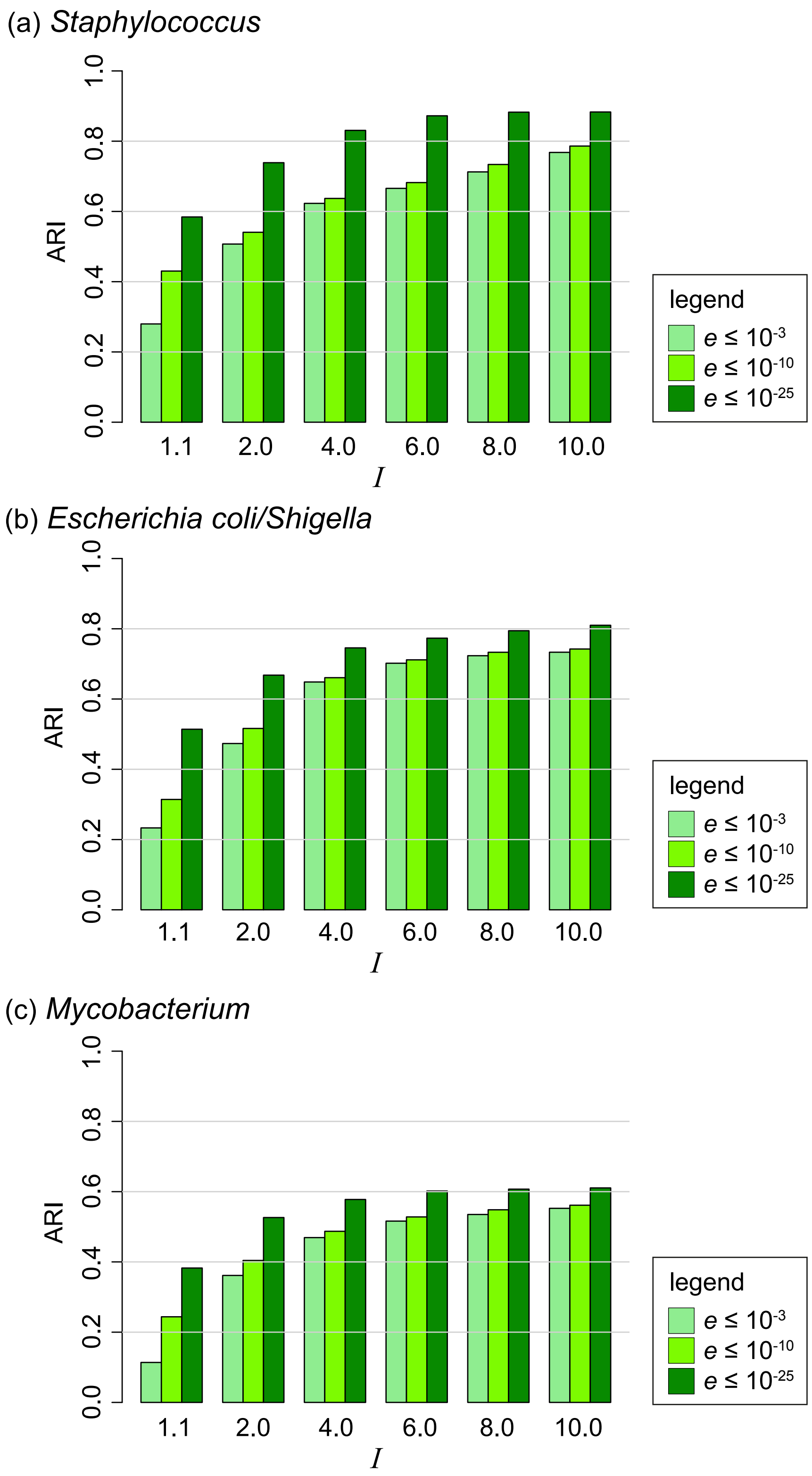

Figure S3. Clustering accuracy of BLAST+MCL across different *e*-value thresholds in BLAST and inflation parameter *I* in MCL for the proteins of (a) *Staphylococcus*, (b) *Escherichia coli/Shigella* and (c) *Mycobacterium*.

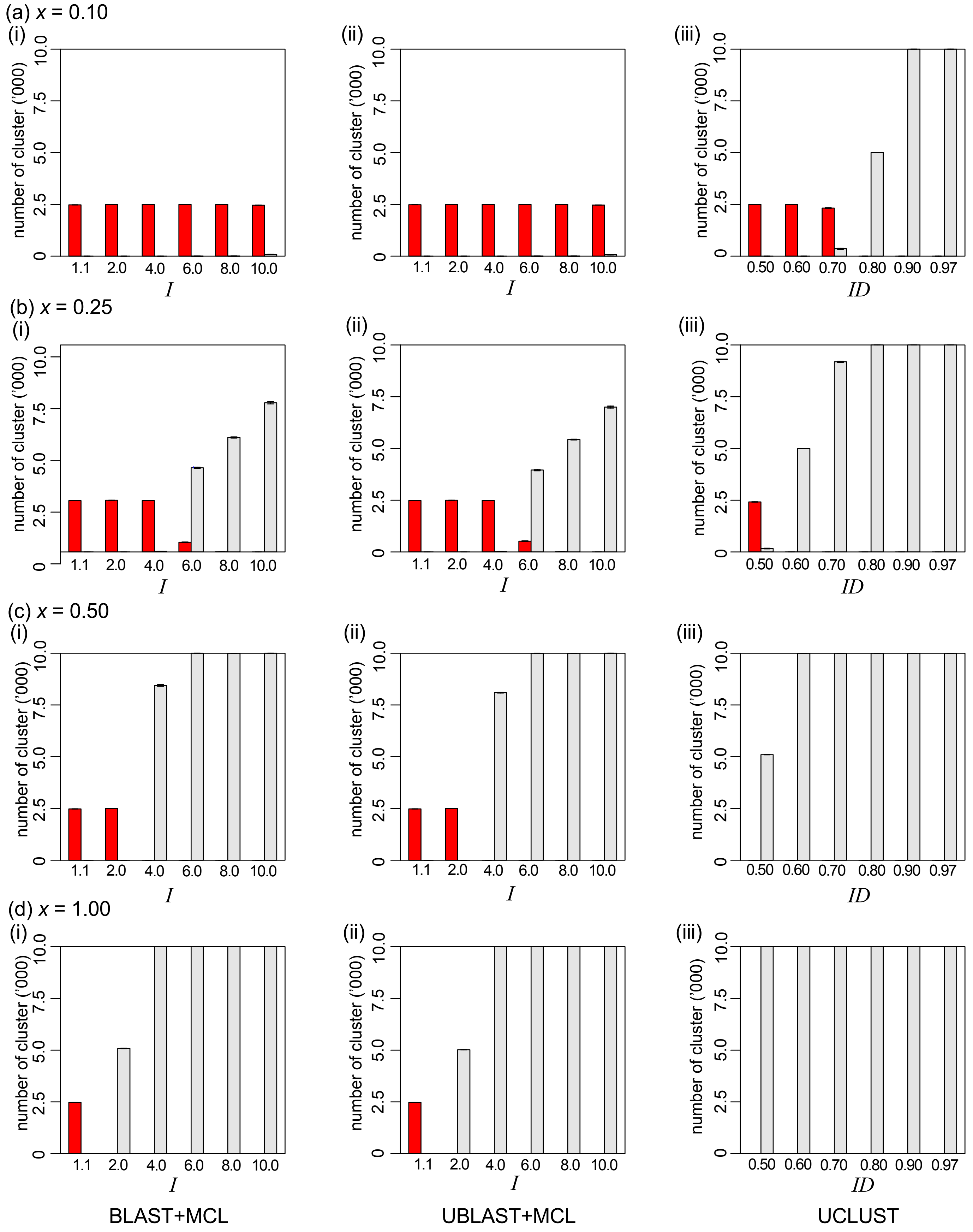

Figure S4. Number of clusters generated across simulated dataset of various divergence levels. Data are shown for different branch lengths on a tree (*x* in Figure 2) at (a) 0.10, (b) 0.25, (c) 0.50 and (d) 1.00, for (i) BLAST+MCL (ii) UBLAST+MCL and (iii) UCLUST, across the specific parameter settings (*I* for MCL; *ID* for UCLUST). The proportion of clusters with size $N \geq 4$ is shown in red bars.

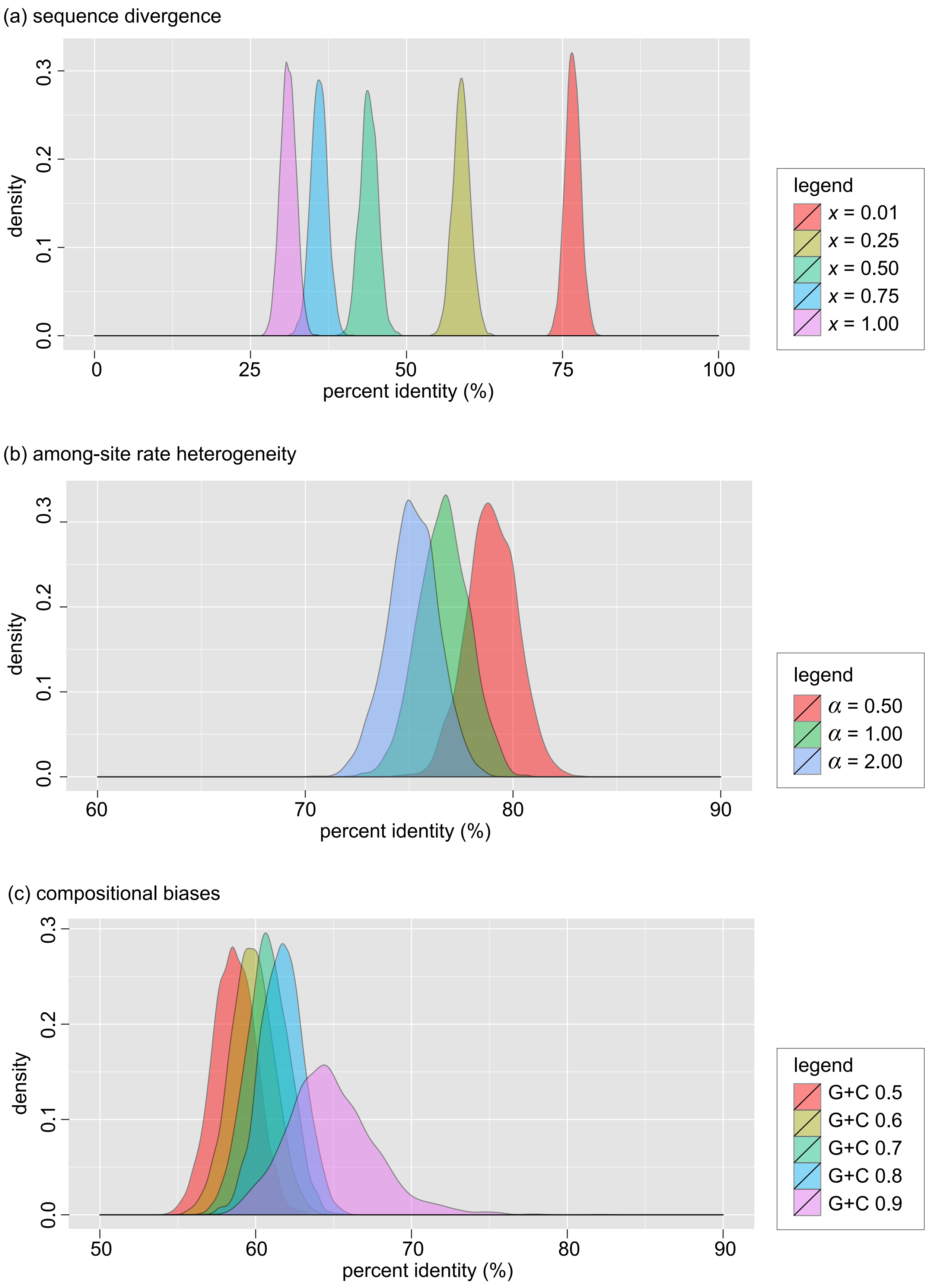

Figure S5. Density histograms of within-cluster sequence similarities across all simulated dataset at various levels of (a) sequence divergence (branch length x in Figure 2), (b) among-site rate heterogeneity (α in gamma distribution) and (c) compositional biases (G+C proportion). Histogram for between-cluster sequence similarities is not shown because almost all (>99.99%) of between-cluster comparisons yielded no significant similarity.

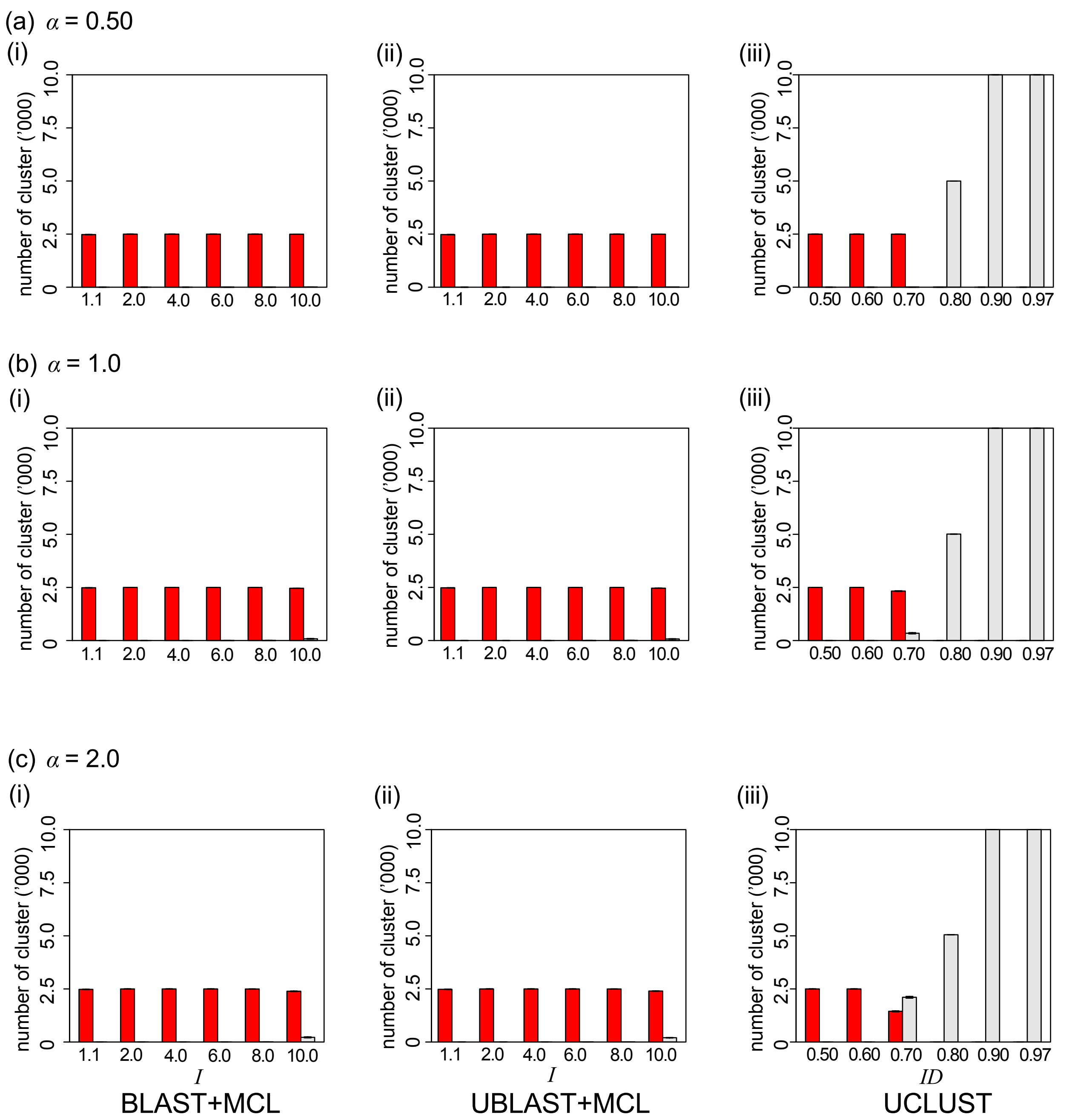

Figure S6. Number of clusters generated across simulated dataset of various rates of heterogeneity. Shown for alpha (α) value in gamma distribution at (a) 0.50, (b) 1.00 and (c) 1.00, for (i) BLAST+MCL (ii) UBLAST+MCL and (iii) UCLUST, across the specific parameter settings (*I* for MCL; *ID* for UCLUST). The proportion of clusters with size $N \geq 4$ is shown in red bars.

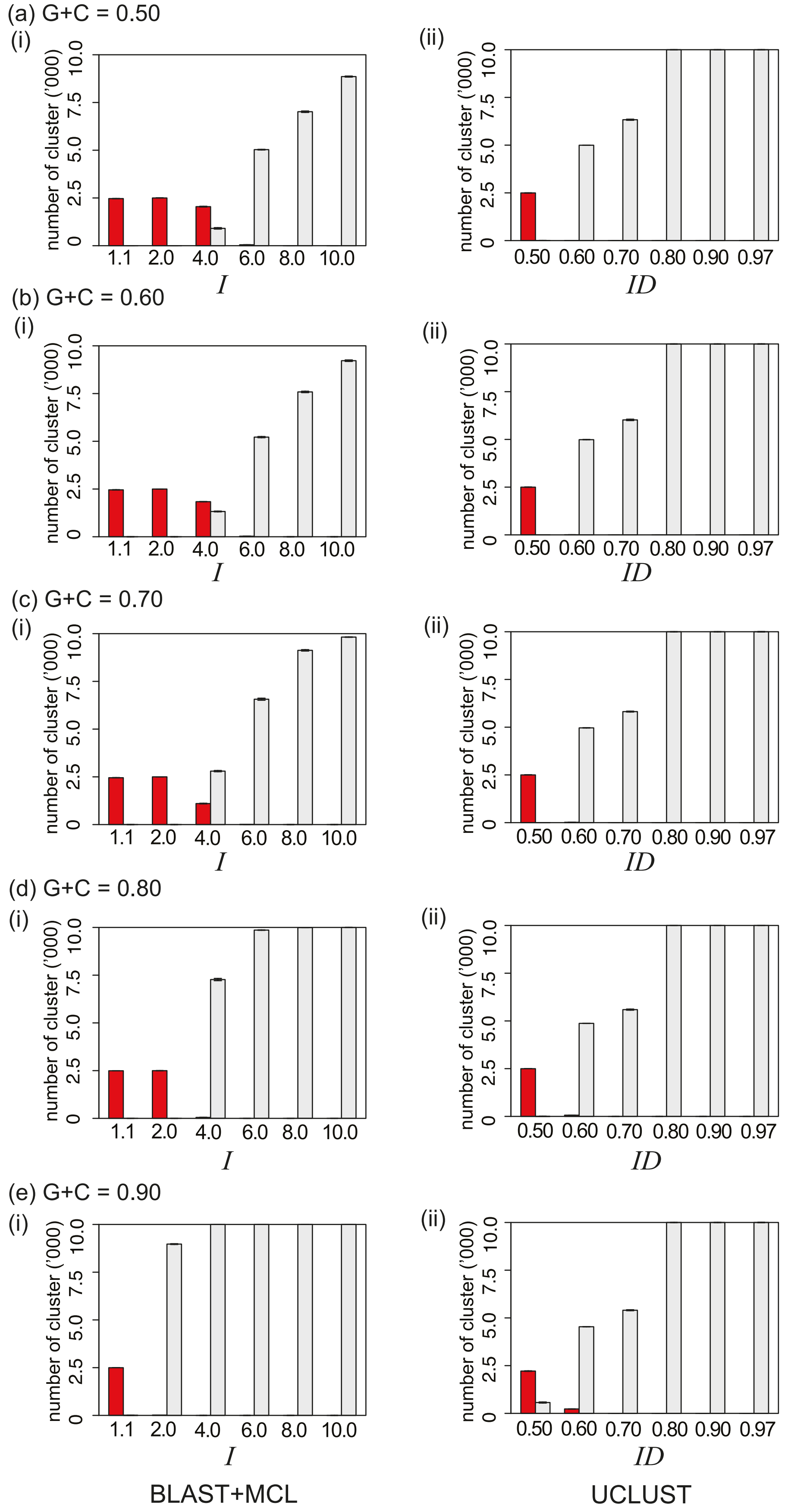

Figure S7. Number of clusters generated across simulated dataset of various G+C portions. Shown for G+C portion at (a) 0.50, (b) 0.60, (c) 0.70, (d) 0.80 and (e) 0.90, for (i) BLAST+MCL and (ii) UCLUST across the specific parameter settings (*I* for MCL; *ID* for UCLUST). The proportion of clusters with size $N \geq 4$ is shown in red bars.

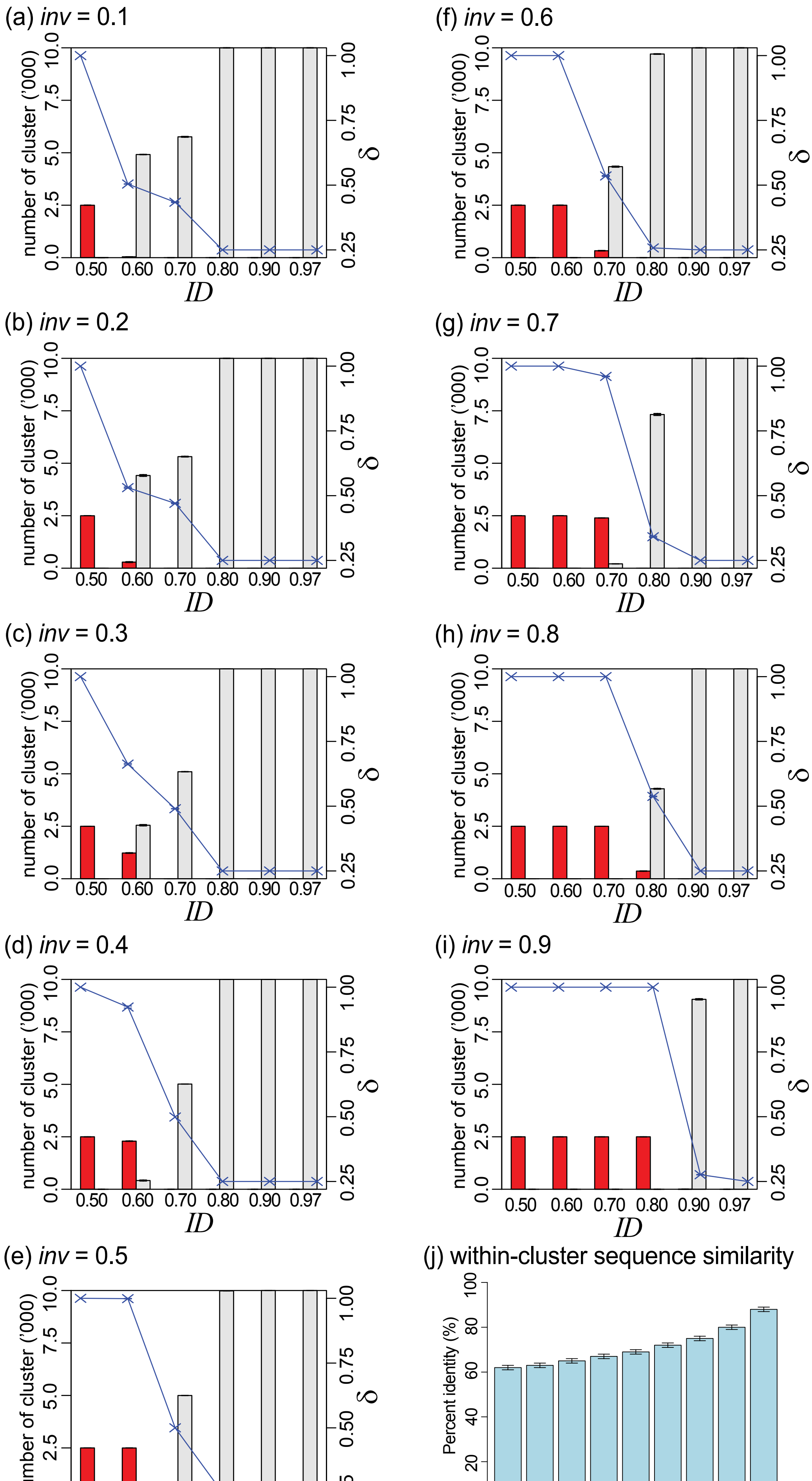

Figure S8. Clustering accuracy of UCLUST across proportion of invariant sites in simulated dataset at 50% G+C. Results are shown for proportion of invariant sites from 0.1 through 0.9 in panels (a) through (i). In each of these panels, the bar chart shows the number of clusters (Y-axis on the left) across different *ID* parameters. All numbers shown are averaged across five replicates in each instance, and the error bars indicate standard deviation from the mean. The proportion of clusters with $N \geq 4$ is shown in red in each bar, and the δ values are plotted within the same panel (Y-axis on the right). Panel (j) shows average within-cluster pairwise sequence similarity of each of these cases.
